# Edpuzzle

**DOI:** 10.5195/jmla.2021.1202

**Published:** 2021-04-01

**Authors:** Erin Ware

**Affiliations:** 1 ware@lsuhs.edu, Reference Librarian, Health Sciences Library, Louisiana State University Health Sciences Center at Shreveport, Shreveport, LA. https://edpuzzle.com/signup/teacher?rc=aok8qa

## DESCRIPTION

Edpuzzle is a simple product that does one thing very well, yet it opens a world of possibilities for users. Edpuzzle just might be the easiest way to create interactive video lessons. It is an e-learning platform that allows users to edit and add questions to literally any YouTube video. Users can also create their own videos and then use Edpuzzle to make them interactive.

Edpuzzle maintains a user-generated content library that allows users to possibly find topics that have already been edited and includes inserted questions. At this time, most of the videos in the content library are focused on K-12 topics; however, this will change as more medical librarians use Edpuzzle.

Edpuzzle allows users to maintain a gradebook on the site. Instructors can construct class lists in Edpuzzle and invite students to create accounts. Instructors can use this list to see which students have completed the assignments, what their answers were, and what they scored. The instructor is also able to see the answers of those students who have not yet finished an assignment.

Multiple-choice, open-ended, and short-answer questions can all be added to videos. Edpuzzle will automatically grade multiple-choice questions. Instructors can also assign videos without adding any questions so they can see which students watch the assigned video and how much of the video they view.

## USES

Librarians can use Edpuzzle in a variety of class settings. In a virtual class, instead of sharing a screen to play a video on how to use the MeSH thesaurus, one could find a National Library of Medicine video on the topic, insert questions into it, and share the link with the students. The instructor can watch students answer the questions in real time and identify anything that was not well understood. In a traditional class setting, a feature called “Go Live” gives the instructor the capability to project the video onto a smartboard while students answer the questions on their computers, laptops, or mobile devices. Alternatively, an instructor could assign the video to be watched before class, then use class time to practice finding MeSH terms and searching with them.

Edpuzzle can also be used to obtain website usage statistics. The open class feature makes it possible to embed interactive videos on LibGuides or websites. Users can then obtain the detailed analytics that Edpuzzle offers.

## TECHNICAL REQUIREMENTS

Edpuzzle is quite simple to use, and since it is web-based, it has no special technical requirements beyond an internet connection and a device capable of playing video. There is nothing that must be installed; however, there is an Edpuzzle browser extension that makes it much easier to find and edit videos directly from YouTube. The browser extension is free and available through the Google Chrome store and Mozilla. Students can use the Edpuzzle app, available for both Apple and Android devices, though this is not necessary.

## COSTS

A basic Edpuzzle account is free. There are also two paid options, a “Pro Teacher” account, which costs $11.50 a month, and a school, district, or university-wide account option that is priced according to the number of users. The free account limits storage space to 20 videos, while a paid account removes the limit; however, users of the free account can increase their storage space by referring others to the service.

## PRIVACY AND ACCESSIBILITY

Edpuzzle is compliant with the Family Educational Rights and Privacy Act (FERPA), the Children's Online Privacy Protection Act (COPPA), and the Web Content Accessibility Guidelines (WCAG) 2.0. Additionally, Edpuzzle can be easily integrated into a learning management system (LMS), such as Google Classrooms, Moodle, or Blackboard.

## COMPARISON

Vizia (vizia.co) is a similar video editing tool that also allows users to add multiple-choice and short-response questions and includes an option to add polls to videos. However, users cannot crop videos with Vizia, nor does Vizia maintain a content library like Edpuzzle. It does not offer the ability to create class lists, but users can choose to require a name and email address to view a video. As with Edpuzzle videos, Vizia videos can be shared by URL or embedded into a website. Viewers of videos created in Vizia can skip watching the videos and go straight to the questions, but with Edpuzzle, the instructor can disable skipping. Both Edpuzzle and Vizia are free to use. Vizia was created by Teachable, which is a course creation and monetization platform.

## CONCLUSION

Librarians or instructors who teach classes, especially virtually, can use Edpuzzle to increase student engagement when using educational videos. The simplicity of use, detailed analytics, and free access make it an especially useful platform for every educator to have in their toolbox.
